# H89 Treatment Reduces Intestinal Inflammation and *Candida albicans* Overgrowth in Mice

**DOI:** 10.3390/microorganisms8122039

**Published:** 2020-12-19

**Authors:** Corentin Dumortier, Rogatien Charlet, Ali Bettaieb, Samir Jawhara

**Affiliations:** 1 UMR 8576-UGSF-Unité de Glycobiologie Structurale et Fonctionnelle, Centre National de la Recherche Scientifique, Institut National de la Santé et de la Recherche Médicale U1285, University of Lille, 59000 Lille, France; corentindumortier59@gmail.com (C.D.); charlet-rogatien@hotmail.fr (R.C.); 2 Faculté de Médecine, Pôle Recherche, University of Lille, 59000 Lille, France; 3 Laboratory of Immunology and Immunotherapy of Cancers, EPHE, PSL Research University, 75000 Paris, France; ali.bettaieb@u-bourgogne.fr; 4 Laboratory of Immunology and Immunotherapy of Cancers (LIIC), EA7269, University of Burgundy Franche-Comté, 21000 Dijon, France; 5 Université de Lille Faculté de Médecine, Pôle Recherche, 1 place Verdun, 59000 Lille, France

**Keywords:** H89, *Candida albicans*, *Escherichia coli*, *Enterococcus faecalis*, *Lactobacillus johnsonii*, microbiota, DSS, colitis, protein kinase A

## Abstract

Deregulation of the dynamic crosstalk between the gut microbiota, intestinal epithelial cells, and immune cells is critically involved in the development of inflammatory bowel disease and the overgrowth of opportunistic pathogens, including the human opportunistic fungus *Candida albicans*. In the present study, we assessed the effect of N-[2-(p-bromocinnamylamino)ethyl]-5-isoquinolinesulfonamide (H89), a protein kinase A inhibitor, on the migration of macrophages to *C. albicans* through dextran sulphate sodium (DSS)-challenged Caco-2 cells. We also investigated the impact of H89 on intestinal inflammation and *C. albicans* clearance from the gut, and determined the diversity of the gut microbiota in a murine model of DSS-induced colitis. H89 reduced the migration of macrophages to *C. albicans* through DSS-challenged Caco-2 cells. In addition, H89 decreased *C. albicans* viability and diminished the expression of pro-inflammatory cytokines and innate immune receptors in macrophages and colonic epithelial Caco-2 cells. In mice with DSS-induced colitis, H89 attenuated the clinical and histological scores of inflammation and promoted the elimination of *C. albicans* from the gut. H89 administration to mice decreased the overgrowth of *Escherichia coli* and *Enterococcus faecalis* populations while *Lactobacillus johnsonii* populations increased significantly. Overall, H89 reduced intestinal inflammation and promoted the elimination of *C. albicans* from the gut.

## 1. Introduction

*Candida albicans* is the most common cause of nosocomial fungal infections threatening patients in intensive care units, immunocompromised patients, and patients with dysfunctional intestinal epithelial barriers [1,2]. *C. albicans* interacts with host cells at the level of the cell wall, which consists mainly of glycans associated with lipids and proteins [3].

The external layer of the cell wall is composed mainly of phosphopeptidomannan and phospholipomannan and the internal layer contains a dense network of polysaccharides consisting of glucans (β-1,3 and β-1,6 linked glucose) and chitin (a polymer of β-1,4-linked N-acetylglucosamine) [4]. Different clinical and experimental studies have shown that an abundance of this fungus correlates with serological markers of Crohn’s disease (CD), namely anti-*Saccharomyces cerevisiae* antibodies (ASCA) [5,6]. CD patients and their first-degree healthy relatives are more frequently and more heavily colonized with *C. albicans* than healthy subjects [7]. In addition, the idiopathic presence of ASCA could be correlated with an abundance of *C. albicans* in healthy parents of CD patients [7]. Animal models of irritable bowel disease (IBD) have led to a greater understanding of CD pathogenesis, especially models of murine colitis [8]. Experimental studies have shown that intestinal inflammation induced by dextran sulphate sodium (DSS) promotes *C. albicans* overgrowth in the gut, and, conversely, that *C. albicans* exacerbates intestinal inflammation in mice [6,9].

The predominant medical therapies for IBD are aminosalicylates, steroids, and anti–tumour necrosis factor (TNF) agents (infliximab and adalimumab), which are effective but are associated with significant side-effects [10,11]. Recently, there has been great interest in the role of cAMP and protein kinase A (PKA) in IBD [12,13]. Zimmerman et al. showed that elevated cAMP levels and increased activation of PKA lead to decreased intestinal epithelial healing and to barrier dysfunction in the intestine [12]. Inactivating cAMP and PKA down-regulated the release of pro-inflammatory cytokines in the mucosa of IBD patients and decreased intestinal inflammation [13]. Kinase inhibitors are now in preclinical development for the treatment of inflammatory diseases [14]. Among these kinase inhibitors, N-[2-(p-bromocinnamylamino)ethyl]-5-isoquinolinesulfonamide) (H89) is a powerful inhibitor of cAMP-dependent PKA [14,15]. The anti-inflammatory effect of H89 has been shown in different in vitro studies, but its anti-inflammatory potential in vivo has not been reported to date.

In the present study, we assessed the effect of H89 on the migration of macrophages to *C. albicans* through DSS-challenged Caco-2 cells. Modulation of cytokine and innate immune receptor expression was evaluated in macrophages pretreated with H89. We also assessed whether treatment of mice with H89 could attenuate the development of colitis and affect the amount of *C. albicans* in mice with DSS-induced colitis. We also measured inflammatory parameters and changes in cultivable bacterial populations in the gut.

## 2. Methods

### 2.1. Cell Line and C. albicans Culture

Colonic epithelial Caco-2 cells were grown in Dulbecco′s modified Eagle medium (DMEM) (Sigma-Aldrich, St. Louis, MO, USA), supplemented with 20 % foetal bovine serum (FBS), 50 IU/mL penicillin and 50 IU/mL streptomycin, at 37 °C and 5% CO_2_. Human monocytic THP-1 cells were incubated in RPMI 1640 medium (Gibco by LifeTechnologies™, Waltham, MA, USA) supplemented with 10% FBS (Gibco, Waltham, MA, USA), 50 IU/mL penicillin and 50 IU/mL streptomycin. THP-1 cells were differentiated into macrophages in the presence of phorbol-12-myristate 13-acetate (25 nmol/L; Sigma-Aldrich, France) at 37 °C and 5% CO_2_. *C. albicans* strain SC5314 was used for this study and was maintained in yeast peptone dextrose broth (1% yeast extract, 2% peptone, 2% dextrose) at 4 °C. To prepare the yeast suspension, *C. albicans* cells were cultured in Sabouraud dextrose broth (Sigma-Aldrich, St. Quentin Fallavier, France) for 24 h at 37 °C in a rotary shaker.

### 2.2. Expression of Pro-Inflammatory Cytokines in DSS-Challenged Caco-2 Cells and Macrophages

Caco-2 cells (10^6^ cells) were placed in DMEM containing 1.5% DSS for 24 h in order to induce epithelial changes. At the same time, H89 was added at a concentration of 10 μM. Caco-2 cells without DSS challenge were used as a control. After incubation, the cells were washed twice in phosphate-buffered saline (PBS) and recovered in RA1 buffer to perform mRNA extraction followed by RT-PCR and q-PCR to analyze TNFα and IL-8 expression [16,17]. For the expression of pro-inflammatory cytokines in macrophages, THP-1 cells (10^6^ cells) were differentiated into macrophages by the addition of phorbol for 72 h. The macrophages were then incubated for 24 h in RPMI medium. Lipopolysaccharide (LPS) was then added to the macrophages at a concentration of 250 ng/mL (LPS from *Escherichia coli* O111:B4; Sigma, L2630-25MG) for 6 h allowing an increase in expression of pro-inflammatory cytokines and some receptors involved in inflammatory signalling pathways. H89 was added to the macrophages at a concentration of 10 μM. After incubation, the macrophages were washed twice with PBS and then treated with RA1 buffer to perform mRNA extraction followed by RT-PCR and q-PCR to analyze IL-1β, IL-6, TNFα, MyD88, NF-kB, TLR2, TLR4 and TLR8 expression. mRNA quantification is described in detail elsewhere [16,17]. Briefly, total RNA was isolated from the macrophages or Caco-2 cells using a commercial kit (Nucleospin RNA/Protein; Macherey-Nagel, Düren, Germany). RNA was measured using a Nanodrop spectrophotometer (Nyxor Biotech, Paris, France) [18]. Fast SYBR green was employed in PCR to amplify the cDNA in a one-step system (Applied Biosystems). The expression was then normalized to the reference gene POLR2A [17,18].

### 2.3. Migration of Macrophages to C. albicans through DSS-Challenged Caco-2 Cells

Caco-2 cells (10^6^ cells) were placed in inserts in the presence of DMEM for 24 h. DMEM containing 1.5% DSS was then added to the Caco-2 cells for 24 h [19]. The Caco-2 cells were pretreated with H89 at a concentration of 10 μM. In parallel, THP-1 cells were differentiated into macrophages using phorbol for 72 h. After washing, the macrophages were maintained in RPMI for 24 h [20,21]. The macrophages were then detached using trypsin and labelled with calcein. Macrophages (10^5^ cells) were then added to each insert. RPMI (150 μL) containing 10^5^ *C. albicans* yeast cells was then added to the lower chamber and the plates were placed in a humidified incubator at 37 °C and 5% CO_2_ overnight. After an overnight migration, non-migrated macrophages were removed from the upper chamber of the trans-well inserts and the migrated macrophages present on *C. albicans* cells were assessed by measuring fluorescence using a fluorometer (FLUOstar^®^ Omega; BMG Labtech). The migration of macrophages through Caco-2 cells unchallenged with DSS (controls) was assigned a value of 100%. This value of 100% corresponds to a healthy intestinal barrier and to intestinal Caco-2 cells unaffected by inflammation while the value 200% corresponds to a destroyed intestinal barrier since the Caco-2 cells are completely damaged. The values obtained during treatment of intestinal Caco-2 cells with DSS (group D) should be between 100 and 200% to ensure that the intestinal barrier is slightly damaged but not destroyed.

### 2.4. The Effect of H89 on C. albicans

For the viability assays, we used a transformant of *C. albicans* expressing luciferase, that has been described in detail elsewhere [14]. The bioluminescent *C. albicans* strain was suspended in PBS at a volume of 10^5^ cells/well (96-well black plates, Chimney well). H89 was then added at their final concentrations (10 μM or 100 μM). Coelenterazine, which is a bioluminescent substrate for luciferase, was then added to the wells at a concentration of 2 μM. Bioluminescence kinetics were then measured (at 30 and 90 min) and analyzed with a luminescence plate reader (FLUOstar Omega Fluorometer, BMG Labtech, Champigny sur Marne, Champigny-sur-Marne, France). The positive control consisted of *C. albicans* strain alone without treatment. In parallel, we assessed the viability of *C. albicans* by culture plate assay. *C. albicans* strain SC5314 was suspended in PBS at a concentration of 10^5^ cells and H89 was then added (final concentration 10 μM or 100 μM). Caspofungin was used as a control at a minimum inhibitory concentration (8 µg/mL). Serial dilutions ranging from 10^−1^ to 10^−4^ were performed on the samples suspended in PBS and 100 μL of each dilution was spread onto Sabouraud agar at 37 °C.

### 2.5. Animals

Female mice from the inbred C57BL/6 strain (3–4-month-old wild-type), certified disease-free (Janvier Laboratories, France) were used in the present study. The use of female mice is based on our previous studies and others that validate this approach [6,8,17]. The mice were housed in the animal facility of the Faculty of Medicine, Lille. The room temperature was maintained at 21 °C and the mice had free access to water and food with exposure to light 12 h/day. All animal experiments were approved by the subcommittee for Research Animal Care, Regional Hospital Centre, Lille, France, and in accordance with institutional (86/609/CEE) and European guidelines for the care and use of laboratory animals. Each mouse was tagged and the mice were separated into seven groups. The control groups comprised: CTL group, receiving water only; Ca group, receiving *C. albicans* only; and H89 group, receiving H89 treatment. The experimental groups were: D (group treated with DSS only), which represented the control group for intestinal inflammation; DCa (DSS + *C. albicans*), which corresponded to the control group for fungal colonization and intestinal inflammation; DH89 (DSS +H89), group to assess the effect of H89 treatment on intestinal inflammation; and DCaH89 (DSS + *C. albicans* +H89), group of interest.

### 2.6. Inoculum Preparation and Induction of Colitis

Mice were given 10^7^ live *C. albicans* yeast cells in 300 µL PBS by oral gavage on day 1. All animals were also given DSS at a concentration of 1.5% (36–50 kDa; MP Biomedicals, LLC, Eschwege, Germany) in drinking water in order to induce colitis. DSS was given to mice for 14 days and was not administered in repeated cycles, since the administration of DSS at a low concentration to mice induces chemical injury to the epithelial lining which mimics the mucosal injury observed in patients with ulcerative colitis [22,23]. For H89 treatment, mice received 5 mg H89/kg/mouse (Sigma-Aldrich, France) in 200 µL PBS daily for 5 days by oral gavage. The dose of 5 mg H89/kg/mouse was established in this in vivo model based on previous publications and on our in vitro migration assay [24,25]. The mice were then sacrificed on Day 14 by cervical dislocation and the colon was collected to determine the fungal load and to carry out PCR and histological examination. In parallel, the stomach, caecum, liver and kidneys were also collected to determine the fungal load. Overgrowth of *C. albicans* in the gut was evaluated by counting the total number of colony forming units (CFU) in 0.1 g/faeces collected from each tagged mouse every 2 days. For quantification of *C. albicans* abundance in faecal samples and in the stomach, ileum, and colon, these samples were cultured on Candi–Select medium, as described in detail elsewhere [6]. For the quantification of cultivable bacteria, colon and faecal samples were cultured on non-selective bacterial media (AC agar) focusing on the most representative cultivable anaerobic and aerobic bacteria that can undergo changes during intestinal inflammation. Mouse faecal samples were collected and serial dilutions were performed daily. These serial dilutions ranged from 10^−1^ to 10^−5^ and were performed on samples suspended in PBS; 100 μL of each dilution was spread onto agar plates. Isolation of aerobic and anaerobic bacteria was carried out on MacConkey and bile esculin azide culture media, respectively [16,26]. For the anaerobic bacteria, the plates were incubated in anaerobic jars containing an anaerobic atmosphere generator pack (AnaeroGenTM 2.5 L; Sigma-Aldrich) at 35 °C. Fluconazole (60 mg/L; Fresenius Kabi, Sevres, France) was added to all aerobic and anaerobic media to inhibit the growth of fungal cells. All bacterial plates were incubated at 37 °C and read after 24 h and 48 h. Identification of bacteria isolated on selective media was performed by MALDI-TOF mass spectrometry (Maldi-TOF; Microflex-Bruker Daltonics, Leipzig, Germany) [16,26].

### 2.7. Clinical and Histological Scores for Inflammation

Clinical scores were evaluated independently, as described previously, by two investigators blinded to the study protocol [8,26]. The body weight of each tagged mouse was recorded daily and the stool consistency and presence of blood in the rectum were also assessed (Table 1) [27]. Three scores (body weight, behaviour, stool consistency and bleeding) were added together, resulting in a total clinical score ranging from 0 (healthy) to 6 (maximum colitis activity) [28]. For the histological score, the colon samples from each mouse were fixed overnight in 4% paraformaldehyde-acid. After fixation, the colon samples were embedded in paraffin, cut in a microtome (4 µm thick; Leica RM2245, Leica Biosystems, Wetzlar, Germany) and affixed onto slides for histological staining. Periodic acid–Schiff stain (Merck, France) was used to stain the colon sections. The colon samples were scanned with a digital slide scanner (Axio-Scan.Z1; Zeiss, Jena, Germany). Histological scoring was carried out by two independent investigators blinded to the study protocol (Table 2). Two scores (infiltration of inflammatory leukocytes into the submucosa and epithelial damage of the colon) were added together and ranged from 0 (no changes) to 6 (extensive cell infiltration and tissue damage) [16,27].

## 3. Statistical Analysis

Differences between groups were analyzed using the Mann–Whitney U test and *p* value of was considered statistically significant, when the *p* value was as follows: *p* < 0.05; *p* < 0.01; *p* < 0.001. All the statistical analyses were performed using XLSTAT (Addinsoft, NY, USA) and GraphPad Prism version 6 (GraphPad, La Jolla, CA, USA).

## 4. Results

### 4.1. Pre-Treatment of DSS-Challenged Caco-2 Cells with H89 Reduces Pro-Inflammatory Cytokine Expression

**Figure 1:**
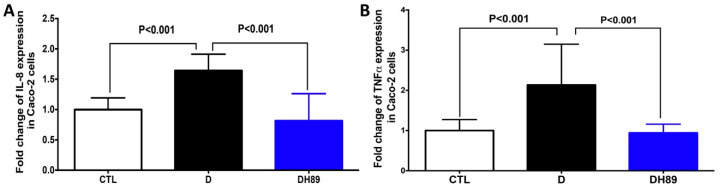
Effect of H89 on the modulation of pro-inflammatory cytokine expression in DSS-treated Caco-2 cells. (**A**,**B**) Relative expression levels of IL-8, (**A**) and TNFα, (**B**) mRNA in Caco-2 cells challenged with 1.5% DSS. CTL corresponds to a control group (Caco-2 cells alone). D corresponds to Caco-2 cells treated with 1.5% DSS. DH89 represents Caco-2 cells pre-treated with H89 and challenged with DSS. The results were obtained from three independent experiments.

Caco-2cells, a well-established human intestinal epithelium model, have been employed extensively in different experimental studies for their interaction with pathogen-derived particles during inflammatory conditions and their ability to produce inflammatory factors including IL-8 and TNFα [29]. To investigate whether H89 can modulate the expression of pro-inflammatory cytokines in Caco-2 cells treated with DSS, we measured the expression of IL-8 and TNFα in this intestinal epithelium model (Figure 1). A significant increase in expression of these two cytokines was observed when Caco-2 cells were challenged with 1.5% DSS for 24 h compared to control conditions (Caco-2 alone). In contrast, a significant decrease in the expression of both TNFα and IL-8 was observed in DSS-challenged Caco-2 cells treated with H89 when compared to DSS-challenged Caco-2 cells alone.

### 4.2. H89 Modulates the Expression of Pro-Inflammatory Cytokines and Toll-Like Receptors (TLRs) in Macrophages

**Figure 2:**
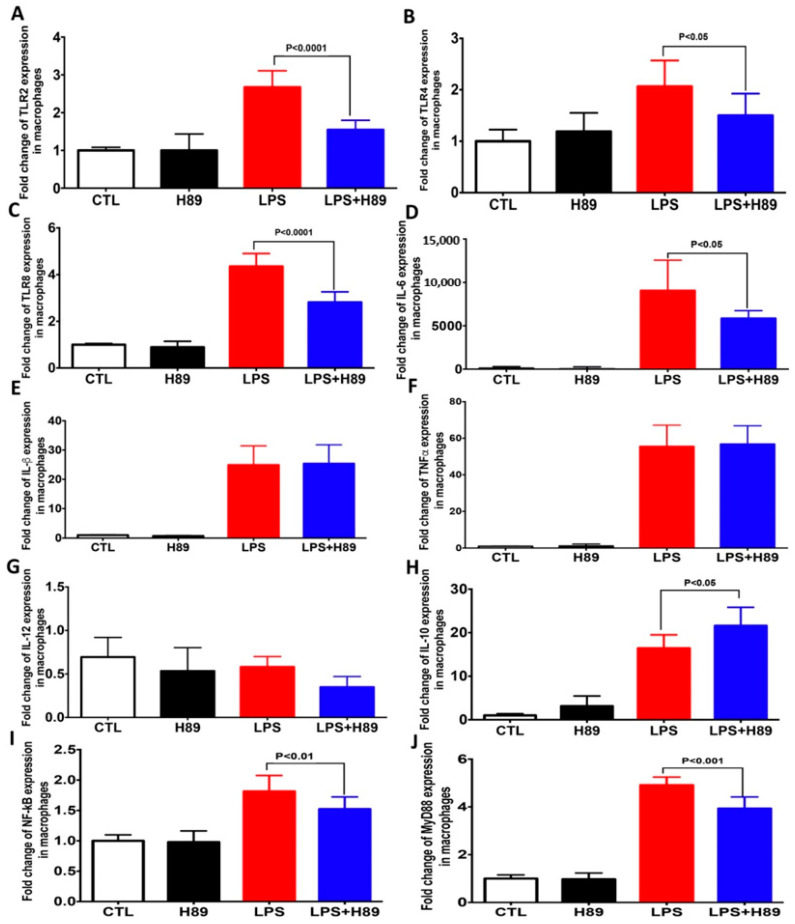
Expression of pro-inflammatory cytokines, signaling pathways and TLRs in macrophages pretreated with H89 and exposed to LPS. (**A**–**J**) Relative expression levels of TLR2, TLR4, TLR8, IL-6, IL-1β, TNFα, IL-12, IL-10, NF-κB, and MyD88 mRNA, respectively, in macrophages. CTL corresponds to a control group (macrophages alone), H89 represents macrophages treated with H89. LPS corresponds to macrophages exposed to lipopolysaccharide. LPS + H89 represents macrophages pretreated with H89 and exposed to LPS.

During intestinal inflammation, macrophages infiltrate the intestinal mucosa and contribute to inflammatory cytokine production [30,31]. To assess the role of H89 in the modulation of macrophage activation, we evaluated the expression of pro-inflammatory cytokines and innate immune receptors in macrophages pretreated with H89 and then exposed to LPS (Figure 2). A significant increase in expression of TLR2, TLR4, and TLR8, and pro-inflammatory cytokines, including IL-1β, IL-6, and TNFα, was observed when macrophages were exposed to LPS compared to unexposed macrophages (controls). Pretreatment of macrophages with H89 (at a concentration 10 μM) in the absence of LPS did not produce any variations in the expression of pro-inflammatory cytokines and TLRs. Macrophages pretreated with H89 and exposed to LPS showed a significant decrease in expression of TLRs (TLR2, TLR4 and TLR8), MyD88, NF-kB as well as IL-6 and an increase in expression of IL-10 while the expression of TNFα and IL-1β did not change (Figure 2).

### 4.3. H89 Decreases Macrophage Migration to C. albicans through DSS-Challenged Caco-2 Cells

**Figure 3:**
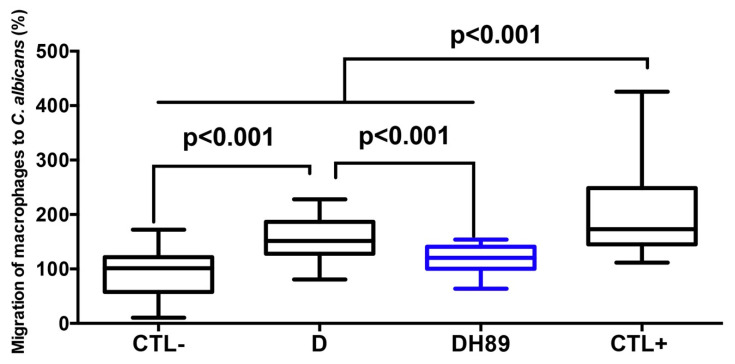
Migration of macrophages pretreated with H89 to *C. albicans* through DSS-challenged Caco-2 cells. CTL- corresponds to a control group (migration of macrophages through Caco-2 cells unchallenged with DSS). D represents migration of macrophages through Caco-2 cells challenged with DSS. DH89 corresponds to migration of macrophages through Caco-2 cells treated with H89 and challenged with DSS. CTL+ represents a positive control (migration of macrophages through totally destroyed Caco-2 cells). The results were obtained from three independent experiments.

Disruption of intestinal epithelial barrier function during gut inflammation promotes the migration of immune cells including macrophages [30,31]. The effect of H89 on the migration of macrophages to *C. albicans* through DSS-challenged Caco-2 cells was assessed (Figure 3). Caco-2 cells unchallenged with DSS (controls) showed low migration of H89-untreated macrophages to *C. albicans* (this migration of macrophages through Caco-2 cells was assigned a value of 100%). The positive control corresponds to easy migration of macrophages through totally destroyed Caco-2 cells (this migration of macrophages through Caco-2 cells was assigned a value of 200%). A significant increase in the migration of macrophages to *C. albicans* through Caco-2 challenged with 1.5% DSS for 24 h was observed while pretreatment of macrophages with H89 significantly reduced migration to *C. albicans* through DSS-challenged Caco-2 cells.

### 4.4. Antifungal Activity of H89 against C. albicans

**Figure 4:**
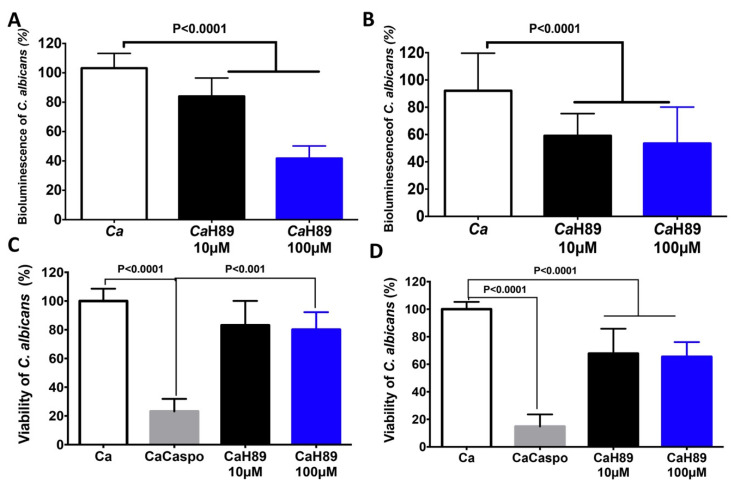
Effect of H89 on *C. albicans*. (**A,B**) Bioluminescence of *C. albicans* challenged with H89 at a concentration 10 μM or 100 μM after 30 and 90 min respectively. The positive control is represented by *C. albicans* alone (Ca). (**C**,**D**), Viability of *C. albicans* by culture plate assay. *C. albicans* challenged with H89 at a concentration 10 μM or 100 μM after 30 min (**C**) and 90 min (**D**). Ca represents a control. CaCaspo corresponds to *C. albicans* challenged with caspofungin. CaH89 represents *C. albicans* challenged with H89.

We determined whether the H89 molecule has an impact on *C. albicans* viability. Bioluminescent *C. albicans* was challenged with H89 at a concentration of 10 μM and 100 μM (Figure 4). We observed a significant decrease in the bioluminescence of *C. albicans* after 30 min of co-incubation with H89 when compared to that of *C. albicans* unchallenged with H89. Additionally, this reduction in bioluminescence was more pronounced after 90 min, at a concentration of 10 μM and 100 μM, indicating that H89 exerts an antifungal activity against *C. albicans* (Figure 4). Additionally, we assessed the viability of *C. albicans* by culture plate assay. H89 significantly reduced *C. albicans* viability and this reduction was more pronounced after 90 min of challenge of *C. albicans* with H89 at a concentration of 10 or 100 µM (Figure 4C,D). The antifungal activities of H89 were not much higher than those of casponfungin, suggesting that H89 exerts antifungal activity against *C. albicans* but is no more efficient than caspofungin (Figure 4C,D).

### 4.5. H89 Decreased Inflammatory Parameters in DSS-Induced Colitis

**Figure 5:**
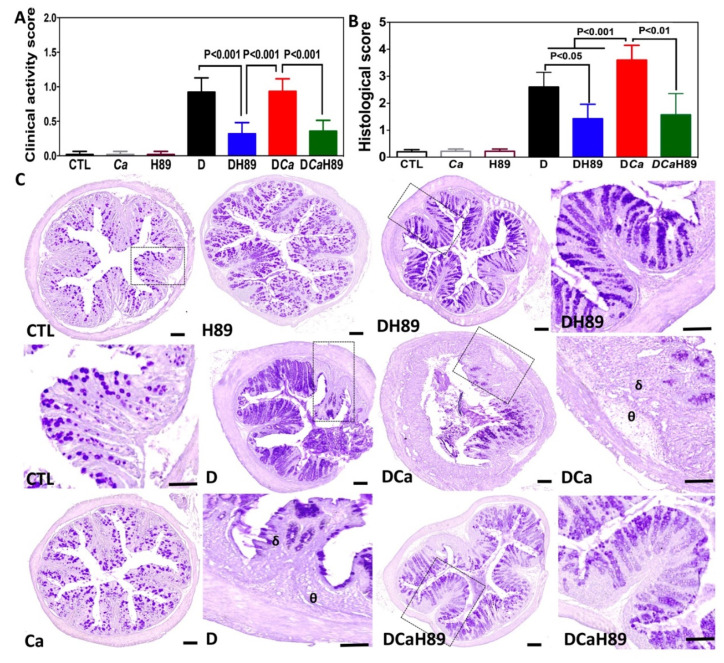
Determination of inflammatory parameters in the DSS-induced colitis model. (**A**,**B**) Clinical and histological scores for inflammation. Control groups correspond to CTL (water), Ca (*C. albicans*) and H89 (mice treated with H89). Experimental groups correspond to D (DSS), DH89 (DSS + H89), DCa (DSS + *C. albicans*) and DCaH89 ((DSS + *C. albicans* + H89). (**C**) Histological analysis of colon sections from DSS-induced colitis. The colon sections (4 µm thick) were stained with periodic acid–Schiff stain. Panels (CTL), (Ca) and (H89) correspond to colon sections from mice receiving water (control), *C. albicans* alone and H89, respectively. Panels (D) correspond to colon sections from mice receiving DSS. Panels (DH89) correspond to colon sections from mice receiving DSS + H89. Panels (DCa) represent colon sections from mice receiving *C. albicans*+ DSS. Panels (DCaH89) represent colon sections from mice receiving *C. albicans* + H89 + DSS. Colon sections from either D or DCa show tissue destruction δ, important inflammatory cell infiltrates and oedema in the mucosa or submucosa of colon wall structures θ. Scale bars represent 10 µm.

To assess whether H89 contributes to the modulation of inflammatory parameters in the DSS-induced colitis model, mice were administered a single oral dose of *C. albicans* and exposed to DSS treatment for 2 weeks in order to induce acute colitis. H89 was then administered orally to the mice for 5 days after *C. albicans* challenge. In terms of the clinical and histological scores for inflammation, control mice (CTL), H89 and Ca (*C. albicans*) groups, did not reveal any inflammatory signs during the whole experiment. In the presence of DSS (D) and DCa (DSS + *C. albicans*), mice showed a significant increase in inflammatory parameters. Although the inflammatory parameters increased during the development of colitis and overgrowth of *C. albicans*, H89 treatment significantly reduced the clinical and histological scores for inflammation when compared to those in the D or DCa groups (Figure 5A,B).

Microscopic observation of colon sections from control groups (CTL, H89 and Ca) did not reveal any alterations to the epithelium, with no inflammatory cell infiltrates in the colon mucosa. Histopathological features in the D and DCa groups showed a high inflammatory process including epithelial damage, scattered inflammatory cell infiltrates in the mucosa and submucosa with the presence of oedema. In contrast, the histopathological changes were less severe in H89 mice than in D or DCa mice and some colon sections showed intact epithelium with no inflammatory cell infiltrates in the colon (Figure 5C).

### 4.6. H89 Decreases the Amount of C. albicans and Restores Anaerobic Bacterial Populations

**Figure 6:**
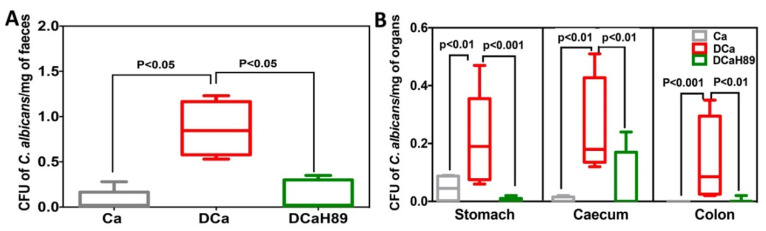
Effect of H89 on *C. albicans* elimination from the gut. (**A**,**B**) Number of *C. albicans* colonies recovered from the stools, stomach, caecum and colon. Data are the mean ± SD of eight mice per group from two independent experiments.

To assess the impact of H89 on the gut microbiota and *C. albicans* overgrowth, the number of *C. albicans* CFUs and the changes in microbiota diversity were determined in freshly collected stool samples from tagged mice, using traditional culture methods based on agar plates (Figure 6).

In the absence of DSS, a significant decrease in *C. albicans* CFUs was recorded in stool samples. Intestinal inflammation induced by DSS promoted *C. albicans* overgrowth in the DCa group and this *C. albicans* overgrowth was correlated with the development of colitis. In contrast, treatment with H89 significantly reduced *C. albicans* in stool samples of DSS-induced colitis mice (Figure 6A). In terms of *C. albicans* overgrowth in the gut, *C. albicans* CFUs were significantly increased in the stomach, caecum and colon of the DCa group while mice treated with H89 showed a significant decrease in *C. albicans* CFUs in all gut segments (Figure 6B).

**Figure 7:**
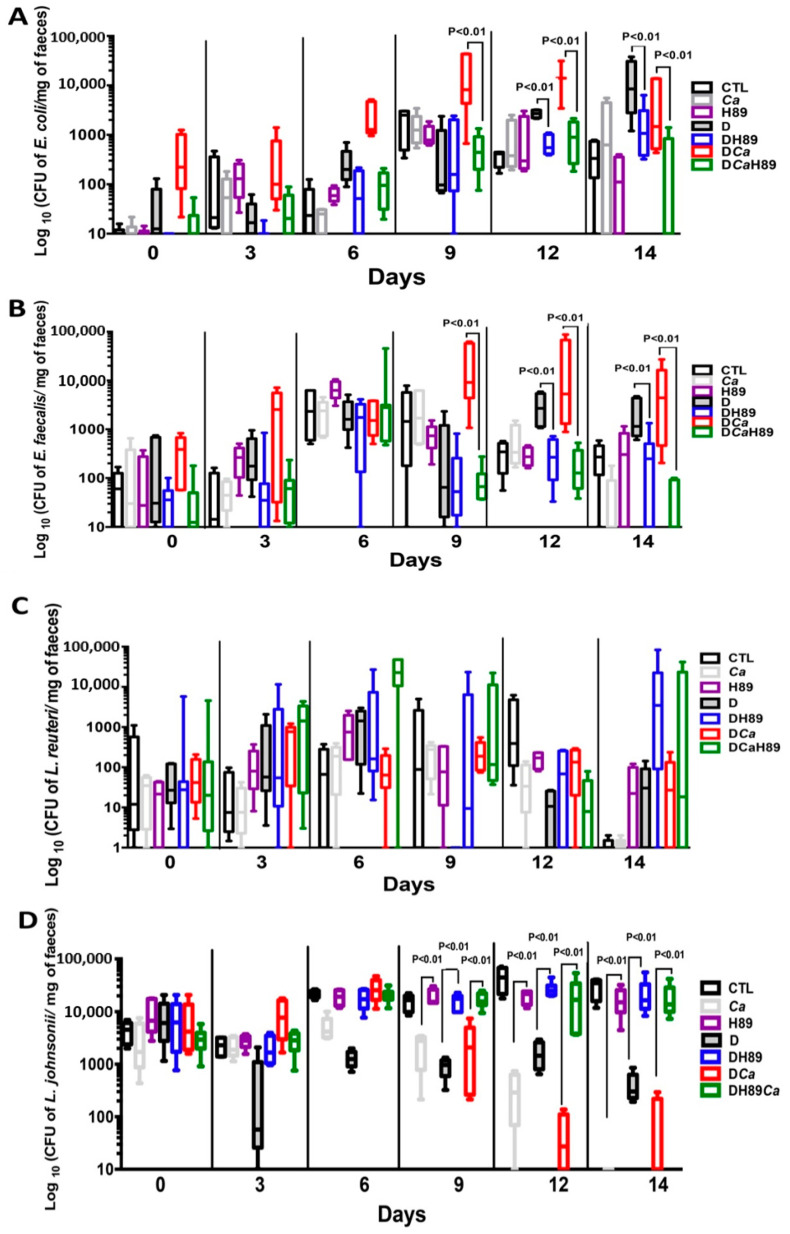
Determination of the number of viable *E. coli* (**A**), *E. faecalis*(**B**), *L. reuteri* (**C**) and *L. johnsonii* (**D**) colonies recovered from stools. For all experiments, stool bacteria were collected from each tagged mouse on day 0 before DSS treatment and *C. albicans* challenge. Data are the mean ± SD of eight mice per group from two independent experiments.

In terms of the changes in aerobic bacteria, *E. coli* and *E. faecalis* populations increased significantly starting from day 9 to day 14 in the D and DCa groups when compared to controls, and this bacterial overgrowth was correlated with the development of colitis. Conversely, a significant decrease in these two bacterial populations was recorded in mice treated with H89 (Figure 7A,B). Similarly, anaerobic bacterial populations, in particular *Lactobacillus johnsonii*, reduced significantly during the development of colitis starting from day 6 (Figure 7). Interestingly, treatment with H89 restored *L. johnsonii* populations. Of note, *Lactobacillus reuteri* populations showed unpredictable changes and an increase starting from day 9 while H89 treatment decreased the fluctuations in *L. reuteri* populations over the course of DSS-induced colitis (Figure 7).

## 5. Discussion

IBDs are chronic, relapsing and destructive disorders that mainly affect the gastrointestinal tract [32]. CD and ulcerative colitis represent two major phenotypic forms of IBD and share some clinical, immunological, and pathological characteristics [32]. Although the exact aetiology of IBD is not well known, it is believed that genetic susceptibility, environmental factors, a dysfunctional immune system and changes to the gut microbiota are involved in the onset and progression of this disease [33]. Different studies have implicated cAMP in inflammatory disorders [12,24,34]. Zimmerman et al. showed that cAMP dysregulates intestinal epithelial cell restitution through PKA indicating that increased levels of cAMP in intestinal epithelial cells inhibit the migratory and restitutive potential in the gut [12]. Additionally, PKA inhibition appears to protect against gut injury in the rat pup model [24]. H89 is an isoquinoline derivative that inhibits PKA by acting as competitive antagonists of ATP at its binding site on the PKA catalytic subunit [35]. Therefore, blockage of ATP binding to PKA prevents cAMP-dependent phosphorylation of PKA substrates [36].

In the present study, we assessed the effect of H89 on the migration of macrophages to *C. albicans* through DSS-challenged Caco-2 cells and how H89 can modulate cytokine and innate immune receptor expression in macrophages and colonic epithelial Caco-2 cells. Macrophages are part of the innate immune system and are instrumental in controlling barrier function in the gut. During intestinal inflammation, leukocytes, including macrophages, can migrate to inflamed colonic mucosa and contribute to secretion of pro-inflammatory cytokines, aggravating tissue damage [37,38]. We observed that pretreatment of macrophages with H89 reduced their migration through DSS-challenged Caco-2 cells, supporting the theory that H89 acts on activation and migration of macrophages into the inflamed site.

In addition, a significant decrease in the expression of TLRs (TLR2, TLR4 and TLR8), MyD88, NF-kB and IL-6 was observed in macrophages pretreated with H89. In line with this observation, a PKA inhibitor suppressed NF-kB-induced breast cancer cell proliferation and multiple NF-kB dependent anti-apoptotic gene expression [39]. Hildebrand et al. showed that H89 diminished LPS-induced release of IL-12p40 in monocytes [40]. In the mouse model of asthma, treatment with H89 inhibited macrophage recruitment in bronchoalveolar lavage fluid [14]. In terms of colonic epithelial Caco-2 cells, we observed that H89 modulated the expression of pro-inflammatory cytokines, in particular TNFα and IL-8, in DSS-challenged Caco-2 cells, and decreased macrophage infiltration and migration indicating that H89 can improve intestinal barrier function. Of note, different investigators have shown that pro-inflammatory cytokines, in particular TNFα, inhibit the absorption of nutrients and the expression of the corresponding transporters in epithelial cells with the involvement of PKC and PKA in this process during intestinal inflammation [41,42].

With regard to *C. albicans*, the cAMP/PKA pathway is involved in morphogenesis, growth, white-opaque switching and fungal virulence in murine models of systemic infection [43,44,45,46]. *C. albicans* PKA contains two catalytic subunits (Tpk1 and Tpk2) that activate downstream targets such as Efg1 and promote various transcriptional regulatory circuits [46]. Bockmühl et al. showed that *C. albicans* deficient in TPK1 and TPK2 displays severe growth defects [44]. Cao et al. reported that inactivation of the PKA catalytic subunit blocked filamentation and dramatically attenuated white-to-opaque switching in *C. albicans* [47]. In line with these observations, the present study shows that H89 reduces *C. albicans* viability, suggesting that H89 may block these two subunits (TPK1 and TPK2) of *C. albicans* PKA.

In DSS-induced colitis model, we assessed whether H89 could attenuate the development of colitis and overgrowth of *C. albicans*. A significant decrease in inflammatory parameters was observed, in terms of clinical and histological score for inflammation in mice treated with H89, supporting the beneficial effect of a PKA inhibitor on the modulation of intestinal inflammation. These data are consistent with in vitro studies showing that H89 attenuates the infiltration of macrophages into Caco-2 cells and decreases the expression of pro-inflammatory cytokines.

In terms of the gut microbiota, the ratio between pathogenic and beneficial bacterial species is disrupted in patients with IBD. A common feature of CD-associated dysbiosis is the decline in microbial diversity, in particular a decrease in the abundance of Firmicutes (e.g., *Lactobacillus* populations) and an increase in Proteobacteria, of which *E. coli* is a member [48,49]. Additionally, enterococci are found in much higher quantities in faecal samples from CD patients [50,51]. This clinical observation is consistent with a high frequency of *E. faecalis* in CD cohorts [51]. Zhou et al. showed that increased *E. faecalis* colonization is associated with clinically active CD [50]. In the experimental mouse model, *E. faecalis* can induce intestinal inflammation, dysplasia and, rectal carcinoma in IL-10 knock-out mice [52]. In the present study, a decrease in *E. coli*, *E. faecalis*, and *C. albicans* populations was observed in mice treated with H89 and *L. johnsonii* populations were restored in these mice. These observations are consistent with our previous studies and other investigators showing that an increased abundance of *E. coli* and *E. faecalis*, and decreased abundance of *L. johnsonii* correlate strongly with increased inflammatory parameters in mice with DSS-induced colitis. Recently, we reported that oral administration of *L. johnsonii* and *Bacteroides thetaiotaomicron* restored the imbalance between aerobic and anaerobic bacterial populations following DSS treatment and *Candida* challenge, and resulted in a significant reduction in inflammatory parameters [53].

In conclusion, H89 reduced the migration of macrophages to *C. albicans* through DSS-challenged Caco-2 cells in a preventive context. In addition, H89 decreased the expression of pro-inflammatory cytokines and innate immune receptors in macrophages and colonic epithelial Caco-2 cells. H89 had antifungal properties against *C. albicans*. In the DSS-induced colitis model, oral administration of H89 not only had a beneficial effect on eliminating *C. albicans* from the gut but also restored the imbalance between aerobic and anaerobic bacterial populations following DSS treatment and *C. albicans* challenge and resulted in a significant reduction in inflammatory parameters revealed by a reduction in clinical and histological scores for inflammation. Overall, these findings provide evidence that H89 treatment attenuated the development of colitis and was involved in the elimination of *C. albicans* from the gut of mice.

## Figures and Tables

**Table 1:** Clinical score for inflammation.

Consistency of Faeces/Blood in Faeces	Score	Behavior	Score	Weight Loss	Score
Normal/No blood	0	Normal	0	None	0
				1–5%	0.5
Wet/Bloody stools or blood around the anus	1	Hunched	1	5–10%	1
				10–15%	1.5
Soft/Severe bleeding	2	Frozen posture	2	>15%	2

**Table 2:** Histological score for inflammation.

Loss of Epithelium and Crypt Damage	Score	Infiltration of Inflammatory Cells	Score
None	0	None	0
0–5%	1	Mild	1
5–10%	2	Moderate	2
>10% loss	3	Severe	3
